# Osteoarthritis in the Knee Joints of Göttingen Minipigs after Resection of the Anterior Cruciate Ligament? Missing Correlation of MRI, Gene and Protein Expression with Histological Scoring

**DOI:** 10.1371/journal.pone.0165897

**Published:** 2016-11-07

**Authors:** Gregor Reisig, Michael Kreinest, Wiltrud Richter, Mechthild Wagner-Ecker, Dietmar Dinter, Ulrike Attenberger, Barbara Schneider-Wald, Stefan Fickert, Markus L. Schwarz

**Affiliations:** 1 Department for experimental Orthopaedics and Trauma Surgery, Orthopaedic and Trauma Surgery Centre (OUZ), Medical Faculty Mannheim, Heidelberg University, Mannheim, Germany; 2 Research Centre for Experimental Orthopaedics, Orthopaedic University Hospital Heidelberg, Heidelberg, Germany; 3 Institute of Clinical Radiology and Nuclear Medicine, University Medical Center Mannheim, Mannheim, Germany; University Hospital Modena and Reggio Emilia, ITALY

## Abstract

**Introduction:**

The Göttingen Minipig (GM) is used as large animal model in articular cartilage research. The aim of the study was to introduce osteoarthritis (OA) in the GM by resecting the anterior cruciate ligament (ACLR) according to Pond and Nuki, verified by histological and magnetic resonance imaging (MRI) scoring as well as analysis of gene and protein expression.

**Materials and Methods:**

The eight included skeletally mature female GM were assessed after ACLR in the left and a sham operation in the right knee, which served as control. 26 weeks after surgery the knee joints were scanned using a 3-Tesla high-field MR tomography unit with a 3 T CP Large Flex Coil. Standard proton-density weighted fat saturated sequences in coronal and sagittal direction with a slice thickness of 3 mm were used. The MRI scans were assessed by two radiologists according to a modified WORMS-score, the X-rays of the knee joints by two evaluators. Osteochondral plugs with a diameter of 4mm were taken for histological examination from either the main loading zone or the macroscopic most degenerated parts of the tibia plateau or condyle respectively. The histological sections were blinded and scored by three experts according to Little et al. Gene expression analysis was performed from surrounding cartilage. Expression of *adamts4*, *adamts5*, *acan*, *col1A1*, *col2*, *il-1ß*, *mmp1*, *mmp3*, *mmp13*, *vegf* was determined by qRT-PCR. Immunohistochemical staining (IH) of Col I and II was performed. IH was scored using a 4 point grading (0—no staining; 3-intense staining).

**Results and Discussion:**

Similar signs of OA were evident both in ACLR and sham operated knee joints with the histological scoring result of the ACLR joints with 6.48 ± 5.67 points and the sham joints with 6.86 ± 5.84 points (p = 0.7953) The MRI scoring yielded 0.34 ± 0.89 points for the ACLR and 0.03 ± 0.17 for the sham knee joints. There was no correlation between the histological and MRI scores (r = 0.10021). The gene expression profiles as well as the immunohistochemical findings showed no significant differences between ACLR and sham knee joints. In conclusion, both knee joints showed histological signs of OA after 26 weeks irrespective of whether the ACL was resected or not. As MRI results did not match the histological findings, MRI was obviously unsuitable to diagnose the OA in GM. The analysis of the expression patterns of the 10 genes could not shed light on the question, whether sham operation also induced cartilage erosion or if the degeneration was spontaneous. The modified Pond-Nuki model may be used with reservation in the adult minipig to induce an isolated osteoarthritis.

## Introduction

Most of the *in vivo* animal models used for the investigation of osteoarthritis (OA) have undergone an intervention in the targeted joint [1,2,3,4]. Changes in the articular cartilage were documented after different periods following the operation as shown in literature after1-14 days [5], 2–6 weeks [6] and 6–52 weeks [7]. Instability should induce changes similar to that in human articular cartilage as described in several publications [8,9,10]. The Pond Nuki [11] model may reflect the degenerative changes in natural OA in dogs and humans [12]. However, the resection of the anterior cruciate ligament (ACL) as starting point of degenerative changes of articular cartilage still remains an injury induced OA [13]. Therefore, development of degenerative changes of articular cartilage is expected in stifle joints after resection of the ACL but not in untreated contralateral joints [6,14]. Development and grading of OA is mainly determined by histological analyses [4,6,14]. In humans, grade and prevalence of OA may be detected by MRI scanning [15,16] and through X-ray examination [17]. Thus, correlation of histological changes and MRI scanning is needed in diagnosis of OA. Emergence of OA is characterized by alterations of gene expression patterns in degenerating cartilage [18,19,20]. That is also true for the animal model of ACLT in the minipig [5].Here the published data address several genes with different behaviour of regulation. Thus, e.g. *col* II may be upregulated according to one [21] but downregulated according to another trial with similar study design [22].

The pig and notably the Göttingen Minipig (GM) is often used in articular cartilage research [5,17,23,24] and is also a common model for the study of regeneration of focal cartilage defects [7,25,26] but the transection of the ACL has rarely been performed in this species up to now [6]. For the articular cartilage research skeletal mature individuals are required as older pigs may mirror the human condition better, as OA is mostly an affliction of older people whether or not there were previous injuries [27,28].

The aim of the study was to induce OA in adult GM by resection of the anterior cruciate ligament (ACLR), verified by histological and MRI scoring as well as analysis of gene and protein expression.

## Material and Methods

### Animals and surgery

Eleven skeletally mature female GM (Faculty of Agricultural Sciences, Animal Breeding and Genetics, Georg-August-Universität, Göttingen, Germany) underwent resection of the anterior cruciate ligament in the left knee. The right knee served as control by sham operation. Two minipigs served as pilot animals. According to the ARRIVE guidelines for reporting animal research [29] we have to notify that one animal was replaced because it had to be killed in accordance with the animal welfare officer due to a very unlikely recovery from an infection of the left knee joint one week after surgery. The life time was 26 weeks.

8 animals were therefore included in our study. Their age was on average 24.75 ± 0.66 months, with a median of 25 months, ranging from 23–25 months. The animal body weights (BW) were on average 55.4 kg ± 9.4 kg, a median of 55.2 kg ranging from 44–67 kg. The study was approved by the ethical committee of the Regierungspräsidium Karlsruhe, Abteilung 3—Landwirtschaft, Ländlicher Raum, Veterinär- und Lebensmittelwesen, Karlsruhe, Germany. The approval has the number: 35–9185.81/G-186/09. The animal welfare officer of the IBF (Interfakultäre Biomedizinische Forschungseinrichtung, Heidelberg, Germany) accompanied the study.

The intramuscular premedication was carried out with azaperon (4 mg/kg; Stresnil, Janssen, Beerse, Belgium), ketamin (10 mg/kg; Ketavet 10%, Vet Way, Elvington, England) and midazolam (1 mg/kg; Midazolam-ratiopharm, Ratiopharm, Ulm, Germany), whereas intravenous application of propofol (1–2mg/kg; Propofol-Lipuro, Braun, Melsungen, Germany) initiated the anaesthesia and allowed the endotracheal intubation. The anesthesia was then maintained with isoflurane (1.5-vol%; Forene®, Abbott, Wiesbaden, Germany) under artificial respiration. Perioperative infection prophylaxis was done by intravenously administered cefazolin (2 g/animal; Basocef Trockensubstanz, Deltaselect, Pfullingen, Germany). Surgery was performed under continuous monitoring of vital parameters and depth of narcosis. After skin incision between patella and tuberosity, the subcutaneous tissue was retraced with the patellar ligament as a key structure. The knee joint was then opened medially of the patellar ligament and the patella was luxated if necessary. Afterwards, the depicted anterior cruciate ligament (ACL) was cut and resected with a hook electrode (Aesculap AG, Tuttlingen, Germany) preventing a reconnection of the parts. Afterwards, closing of the wound was done in layers after lavage. Then the wound was covered by a plaster to prevent dirt from entering the wound. A drawer test was performed to confirm instability by the resection of the ACL. The right knee was equally opened, purged and closed, but no cut/resection took place. Analgesia was performed with fentanyl (0.2 mg/animal; Fentanyl-Janssen, Janssen, Neuss, Germany) and carprofen (4 mg/kg; Rimadyl Rind, Pfitzer, Berlin, Germany). All animals were closely monitored for signs of pain, illness and unusual favouring of the hind legs after operation.

### Housing and sacrifice of the animals

The animals were allowed to acclimatize before the operation procedure. Water was provided *ad libidum*. Full weight bearing and free motion was possible before and after surgery. After the operation, the animals were monitored daily. Carprofen (4 mg/kg; Rimadyl Rind, Pfitzer, Berlin, Germany) was applied when needed. 10 days after surgery, the animals were transferred to a licensed farmer and were cared for the remaining life time. 26 weeks after the operation, the animals were first assessed by MRI and x-ray under anaesthesia (see below) and then killed by intravenous application of 20 mL saturated KCl-solution in deep narcosis under electrocardiographic supervision. Afterwards, a positive drawer sign in the left knee confirmed an inoperable ACL, but there was no positive sign in the right knee.

### Preparing the animals for MRI

As the pilot trials showed, the animals had to be continuously sedated to prevent movements of the animals which would distort the scanning process. Thus, the MRI assessment was performed under general anesthesia with pentobarbital (Narcoren®, Merial GmbH, Halbergmoos, Germany) given continuously i.v. (10mg/kg per hour). The medication was applied via a perfusor (Terufusion TE-311, Terumo, Eschborn, Germany) under artificial ventilation (Servo 300A, Maquet, Rastatt, Germany). Premedication was performed analogous to the ACL-surgery. Furthermore, a central vein catheter was placed into the jugular vein to provide a safe and large venous access [30]. Additionally, a transurethral catheter was placed [30] and the animals were wrapped in diapers to prevent pollution. The assessments were performed under constant monitoring. Soft towel paper was plugged into their ears to reduce noise exposure.

### MRI assessment

On the scheduled day of killing, the MRI-scans were performed consecutively on both knee joints of each minipig. A 3-Tesla high-field magnetic resonance tomography unit (Magnetom Trio, Siemens, Erlangen, Germany) and a 3T CP Large Flex Coil (Siemens, Erlangen, Germany) were used. After a localizing scan, scanning procedures with standard proton-density weighted fat saturated sequence in coronal and sagittal direction, slice thickness 3 mm with 10% gap, adapted from human scanning procedures were applied. The digital scans were then blinded and afterwards assessed by two radiologists who had more than 8 years experience each in musculoskeletal MRI (DD, AU) on an external work station (MacPro, Apple Inc., Cupertin, CA). An FDA approved OsiriX MD Imaging Software was used. The WORMS-score [31] for degenerative signs of the cartilage was adopted. Finally a consensus between the two experts’ assessments was reached.

### X-ray assessment

Both knee joints of each minipig, which were still under anaesthesia, were x-rayed in anterior-posterior position with manually extended limb and in lateral position. The focal distance was 1.3 m and the x-ray tube voltage was 66 kV. The exposure time varied as it was automated by the device (DigitalDiagnost, Philips, Hamburg, Germany). The images were then appraised according to the Kellgren and Lawrence [32] scoring system by one senior physician (MS) and one medical student (MK).

### Sample collection

After killing the pig, the left and right knee of each minipig were opened and macroscopically examined for signs of change. Afterwards, a punch with 4 mm in diameter was used to excise a sample from the medial and lateral condyle and the medial and lateral tibia plateau surface. Each sample was taken from the most affected area or, if no change was macroscopically visible, from the main loading zone. These samples were then fixated in 4% formaldehyde for histology and immunohistochemistry. Now the cartilage in a diameter of 10 mm in the zone around the punched hole was excised down to the bone with a scalpel and stored in liquid nitrogen for later isolation and processing of the RNA. The used instruments were rendered RNAse free by treatment with hydrogen peroxide [33].

### Histology

The formalin-fixed femoral and tibial osteochondral specimens from the joints were washed and decalcified with EDTA (Merck, Darmstadt, Germany) for 3–4 weeks. The samples were then washed to remove EDTA, then automatically (TP1020 Leica, Wetzlar, Germany) dehydrated and afterwards embedded in paraffin with a casting implement (EG1140C Leica, Wetzlar, Germany) for cutting. 5 μm thick slides were sectioned (RM2165 Leica, Wetzlar, Germany) and mounted on Superfrost-Plus glass slides (R. Langenbrick, Emmendingen, Germany). Prior to staining, the slides were deparaffinised with xylene and rehydrated in a graded series of ethanol/water blend. To show overall tissue morphology, the deparaffinised slides were subjected to an adapted hematoxylin/eosin (HE-staining) staining protocol [34] for an unsophisticated assessment of cell and tissue morphology by a light microscope (DMRE Leica, Wetzlar, Germany) with an attached camera (DFC 300 FX Leica, Wetzlar, Germany). We did not aim to perform a statistically based analysis by evaluation of the HE—stained tissue. Furthermore, a safranin-o staining was conducted to evaluate the distribution of proteoglycans in the cartilage and the slides were surveyed with the aforementioned equipment. The slides were blinded, examined and assessed by 3 experts (WR, MW-E, MS) according to the Little-score [35]. Afterwards, a consensus between the 3 experts was reached.

### HE-staining

After deparaffinization and rehydration of the slides, they were stained with Mayers hematoxylin for the duration of 10 minutes (Merck, Darmstadt, Germany) and then washed in tap water for 15 minutes. Now they were stained with eosin (Carl Roth, Karlsruhe, Germany) for 3 minutes, followed by dehydration in an ascending alcoholic concentration. The conservation was carried out with Eukitt (O. Kindler GmbH, Freiburg, Germany).

### Safranin O-staining

The slides were deparaffinised, rehydrated and stained in Weigerts hematoxylin (Carl Roth, Karlsruhe, Germany) for 8 minutes, washed in tap water for 10 minutes, stained with Fast Green (Merck, Darmstadt, Germany) for 5 minutes and differentiated with 1% acetic acid (Merck, Darmstadt, Germany) for 5 seconds. Afterwards, the specific staining of the proteoglycans was done with Safranin-o (Sigma-Aldrich Chemie GmbH, Munich, Germany). Then the slides were dehydrated in an ascending alcoholic concentration and conserved in eukitt (O.Kindler GmbH, Freiburg, Germany).

### Immunohistochemistry

Additionally, the slides were subjected to an immunohistochemistry with COL1A1 (sc59772, Santa Cruz Biotechnology Inc., Heidelberg, Germany) and COL2 (ab3092, abcam, Cambridge, UK) antibodies. After deparaffinization of the slides with xylene and rehydration in a graded series of ethanol/water blend, they were then fixed for 30 min in H_2_O_2_ and afterwards subjected to antigen retrieval (heat induced: 10mM sodium citrate, pH 6.0) for 20 minutes. After they had been washed and then blocked with 2% BSA for 80 minutes, the slides were incubated for 70 minutes with the respective antibody (Dilution 1:100 COL1A1, 1:50 COL2) at room temperature and then washed again. The secondary antibody (Vectastain Universal Elite Kit, Vector Laboratories, Burlingame CA, US) in a 1:100 dilution was now applied for 50 minutes and, after additional washing, the ABC-complex (Vectastain Universal Elite Kit, Vector Laboratories, Burlingame CA, US) was applied for another 30 minutes. After this process the slides were stained with 3,3’-diaminobenzidine (Vector Laboratories, Burlingame CA, US) for 4 minutes in the dark and then washed for 5 minutes in tap water. After dehydration in an ascending alcoholic concentration and xylene, the slides were conserved in eukitt (O.Kindler GmbH, Freiburg, Germany). The individual slides were examined using a light microscope (DMRE Leica, Wetzlar, Germany) with an attached camera (DFC 300 FX Leica, Wetzlar, Germany). The slides were assessed according to their staining with 0 (no staining), 1 (little), 2 (moderate) and 3 (distinct) points (GR).

### Quantitative PCR

The cartilage samples stored in liquid nitrogen were homogenized (POLYTRON PT 3000, Kinematica AG, Lucerne, Switzerland) in TRIzol® (Life Technologies GmbH, Darmstadt, Germany) and the RNA was isolated according to the manufacturer’s instructions. Afterwards, the RNA-samples were cleaned with a mini-kit column (Mini Kit, Qiagen, Hilden, Germany). The cleaned RNA was then transcribed into cDNA (Sensiscript RT Kit, Qiagen, Hilden, Germany). To obtain a standard curve for each gene, cDNA was submitted to a PCR with the specific primers for each gene: collagen 1A1 (*col1A1)*, collagen 2 (*col2)*, aggrecan (*acan)*, interleukin 1ß (*il-1ß)*, matrix metalloproteinase -1 (*mmp1*), -3 (*mmp3)*, -13 (*mmp13)*, Vascular endothelial growth factor (*vegf)*, ADAM metallopeptidase with thrombospondin type 1 motif, 4 and 5 (*adamts4* and *adamts5)* and ß-actin (*actb)*; (primer sequences see Table 1). The resulting PCR-product was cleaned with an agarose gel followed by gel extraction (QIAquick Gel Extraction Kit, Qiagen, Hilden). Resulting DNA-concentration was measured and ten fold dilutions were conducted for each gene. Afterwards, a quantitative real time PCR (qRT-PCR) was carried out in duplicate for each dilution on a qPCR-System (Mx3005 P Stratagene, Waldbronn, Germany) with the QuantiTect SYBR-Green PCR Kit (Qiagen, Hilden, Germany) according to the manufacturer’s instructions and gene specific annealing temperatures. Thus, a standard curve for each gene was created. Each sample RNA was transcribed as mentioned above and submitted to a qRT-PCR in duplicate, with the housekeeping gene ß-actin as reference [36].

**Table 1:** Sequences of the used primers and their references.

Name	Sequence (F: forward / R: reverse)	PCR-Product size [base pairs]	Author
Collagen 1A1	F: CCAACAAGGCCAAGAAGAAG	64	Gredes et al.
	R: ATGGTACCTGAGGCCGTTCT		2007 [37]
Collagen 2	F: CACGGATGGTCCCAAAGG	102	Steck et al.
	R: ATACCAGCAGCTCCCCTCT		2009 [38]
Aggrecan	F: TTCCCTGAGGCCGAGAAC	194	Zou et al.
	R: GGGCGGTAATGGAACACAAC		2008 [39]
Interleukin 1ß	F: CAGCCATGGCCATAGTACCT	216	Tayade et al.
	R: CCACGATGACAGACACCATC		2006 [40]
MMP 1	F: TCAGTTCGTCCTCACTCCAG	321	Le Graverend et al.
	R: TTGGTCCACCTGTCATCTTC		2002 [41]
MMP 3	F: AATGATCACTTTTGCAGTTCGAGAA	76	Zeni et al.
	R: GGCATGAGCCAAAACTTTTCC		2007 [42]
MMP 13	F: TTGATGATGATGAAACCTGGA	69	Gredes et al.
	R: ACTCATGGGCAGCAACAAG		2007 [37]
VEGF	F: GAGACCAGAAACCCCACGAA	138	Zhou et al.
	R: GCACACAGGACGGCTTGAA		2009 [43]
ADAMTS-4	F: CAGGGTCCCATGTGCAACGT	115	Huang et al.
	R: CATCTGCCACCACCAGGGTCT		2011 [44]
ADAMTS-5	F: TTCGACATCAAGCCATGGCAACTG	197	Huang et al.
	R: AAGATTTACCATTAGCCGGGCGG		2011 [44]
ß-actin	F: CAAGGAGAAGCTCTGCTACG	245	Steck et al.
	R: AGAGGTCCTTCCTGATGTCC		2009 [38]

### Statistical Analysis

Statistical analysis was done using the software SAS 9.3 (SAS, Heidelberg, Germany), Excel 2010 (Microsoft, Redmond, WA, USA) and Origin 8.6.0.G (OriginLab Corporation, Northampton, MA, USA). Correlation was performed between the histological and MRI results, also between the histological and gene expression results and finally, between the histological and immunohistochemical findings. The difference between the results representing the ACLR side and the sham side (right) of the knee was tested using the t-test procedure. The level of significance was 0.05. In the box and whisker plots the grey boxes represent 25% to 75% of the values, the whiskers show the minima/ maxima and the small black square represents the mean, whereas the line in the grey box illustrates the median.

Sample size calculation was done for the study regarding changes in the menisci in the stifle joints, which is described elsewhere [45].

## Results

From the 11 GM that were operated, two served as pilot animals to establish the MRI procedures in particular and one replaced an animal which had to be killed as it was suffering from an infection of the ACLR knee joint.

At the day of their killing the included animals showed normal gait patterns.

The drawer test was performed for all knee joints immediately after the animals had been killed. Whereas all the ACLR knee joints showed a positive drawer sign with higher laxity, the sham knee joints showed a negative drawer sign with a hard stop.

**Fig 1:**
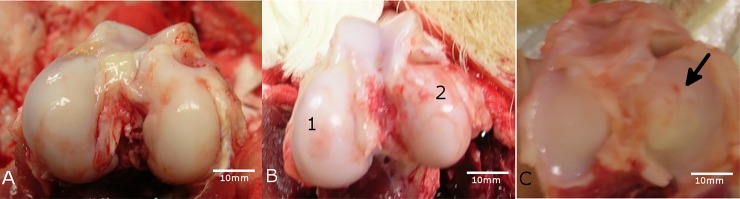
Macroscopic overview. A) The femoral condyles of the ACLR knee joint of animal #8952 showed no visible change/deterioration of the cartilage. B) The femoral condyles of the ACLR knee joint of animal #8955 showed different characteristics in form of a reduced cartilage coating (1) in the lateral condyle and even erosion of the cartilage (2) in the medial condyle. C) The lateral tibia plateau surface of the sham-operated knee joint of animal #8958 showed a slightly rough cartilage surface (arrow). A-C: The scale bars were determined with the corresponding x-ray images using the program RadiAnt DICOM viewer (build 3.4.1.13367, retrieved from http://www.radiantviewer.com; October 12th 2016; Medixant (Poznan, Poland)).

The macroscopical assessment of the tibial and condylar cartilages of the knee joints showed various states of deterioration, from smooth hyaline cartilage layer (no change) up to vanished cartilage with exposition of the subchondral bone (Fig 1A–1C).

**Fig 2:**
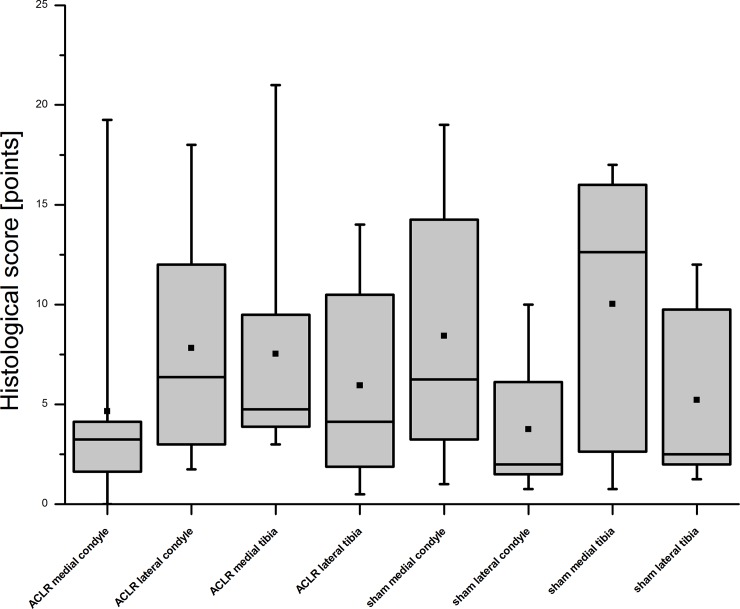
Results of the histological scoring after consensus. The score for each area of interest–the medial/lateral condyle and the medial/lateral tibiaplateau of the ACLR and sham-operated knee joints- of eight Göttingen Minipigs are shown. The used score [35] ranges from 0 points with no changes up to 25 points. As the results of the scoring showed, the sham knee joints were also affected with no significant differences between the ACLR and sham operated knee joints.

**Fig 3:**
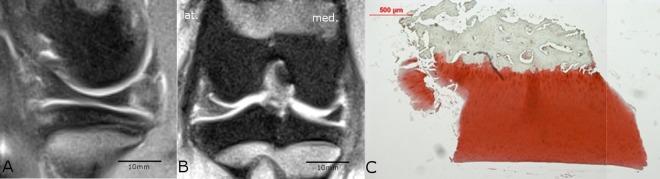
**Comparison of MRI (A and B) with histological findings (C).** The medial condyle region of the ACLR knee joint of animal #8958 showed no salience neither in the sagittal scan through the medial compartment of the knee (A) nor in the coronal MRI-scan (B). The scale bars for A and B were determined with the program RadiAnt DICOM viewer (see caption Fig 1). The histological safranin-o staining (C) showed a normal cartilage tissue (Little-score: 0 points). The histological and MRI results did match.

**Fig 4:**
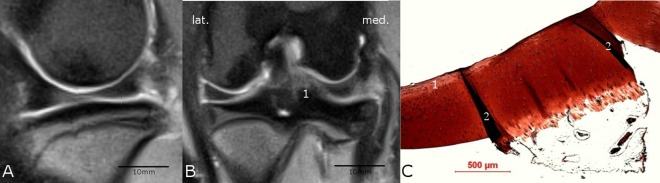
**Comparison of MRI (A and B) with histological findings (C).** The medial tibia plateau region of the ACLR knee joint of animal #8952 showed in the sagittal MRI-scan (A) through the medial compartment no salience but in the coronal MRI-scan (B) a signal irregularity (1) of the normally bright appearing cartilage was present and was therefore rated as pathological; no cyst or osteophytes were present. The scale bars for A and B were determined with the program RadiAnt DICOM viewer (see caption Fig 1). C) The histological safranin-o staining showed a mild degeneration (Little-score: 4.0 points) with only a slight reduction in staining (1). The darker formations in the cartilage (2) were interpreted as creases of the slice and were not included in the scoring. Thus, the histological and MRI findings did not match.

**Fig 5:**
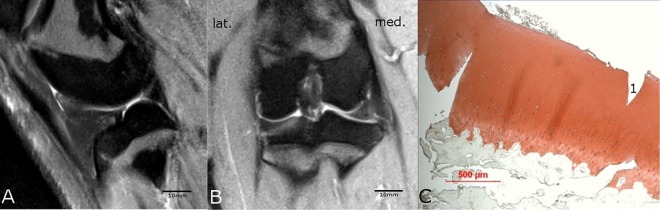
**Comparison of MRI (A and B) with histological findings (C).** The lateral tibia plateau region of the sham knee joint of animal #8958 showed no salience neither in the sagittal MRI-scan (A) through the lateral compartment of the knee nor in the coronal MRI-scan (B) and was therefore rated as unchanged. The scale bars for A and B were determined with the program RadiAnt DICOM viewer (see caption Fig 1). In contrast to the MRI results, the histological safranin-o staining (C) showed a moderate degeneration (Little-score: 10.5 points) with a fissure (1) in the cartilage and detachment of the topmost layer of the cartilage tissue. The histological finding and the MRI result did not concur.

**Fig 6:**
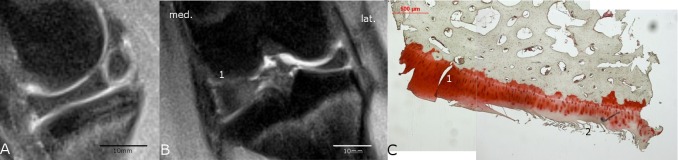
**Comparison of MRI (A and B) with histological findings (C).** The medial condyle region of the ACLR knee joint of animal #8955 showed in the sagittal MRI-scan (A) through the medial compartment no salience but the coronal MRI-scan (B) showed a signal irregularity (1) in the cartilage layer and was therefore rated as pathologically changed. The scale bars for A and B were determined with the program RadiAnt DICOM viewer (see caption Fig 1). The histological safranin-o staining (C) showed a severe degeneration (Little-score: 19.25 points) with a fissure (1) reaching to the subchondral lamella and severe erosion (2) of the cartilage. Reduced safranin-o staining of the remaining cartilage tissue indicated a reduced GAG content and a severe loss of chondrocytes could be seen. The histological and MRI results did match.

The histological analysis of the cartilage samples exhibited variable degeneration grades throughout the examined sites of the knee joints (Figs 2–6).

The changes ranged from none with a smooth surface (Fig 3C) or very small surface irregularities of the cartilage (Fig 4C) to fissures in the cartilage surface (Fig 5C) up to erosion of the whole cartilage (Fig 6C). Also the chondrocyte density varied and a cloning of cells was evident. The interterritorial staining was reduced in some of the samples. The outcome of the histological scoring for each area is presented in Fig 2. The mean score of the ACLR medial condyle sample was 4.66 ± 5.69 points, respectively 7.81 ± 5.48 points for the ACLR lateral condyle. The ACLR medial and lateral tibia plateaus were scored as 7.53 ± 5.66 and 5.94 ± 4.85 points. The sham medial and lateral condyle samples showed a score of 8.44 ±6.20 respectively 3.75 ± 3.11 points. And the sham medial and lateral tibia plateaus displayed 10.03 ± 6.47 and 5.22 ± 4.18 points. No significant differences (p = 0.7953) between the ACLR and sham-op knees were found (6.48 ± 5.67 vs. 6.86 ± 5.84 points).

**Fig 7:**
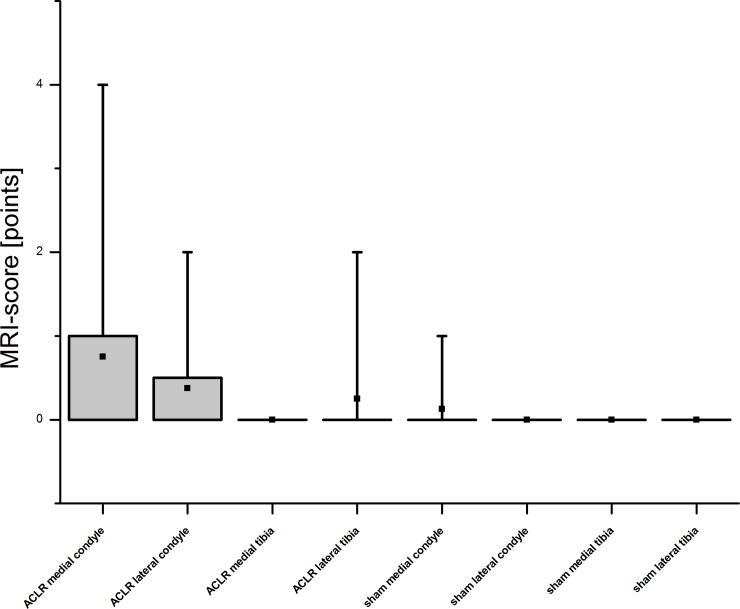
MRI scoring. MRI scans of eight Göttingen Minipigs were scored according to an adapted WORMS-score. The score ranges from 0–22 points. The results showed that in the samples, either from ACLR or sham knee joints, no or only minor changes were visible in the MRI images.

**Fig 8:**
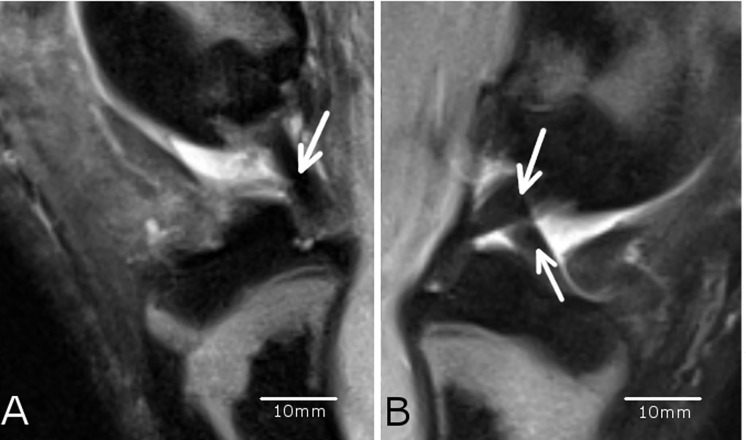
Presence of the ligaments. The ACLR knee joint A) of animal #4085 showed only the *ligamentum cruciatum posterius* (arrow) whereas the sham knee joint B) of the same animal showed both cruciate ligaments were present (arrows). This showed that the operation of resecting the *ligamentum cruciatum anterius* was successful (A). A, B: The scale bars were determined with the program RadiAnt DICOM viewer (see caption Fig 1).

In the MRI examination only few changes of the cartilage were evident (Fig 7) in terms of reduced or even vanished signal strength or other irregularities (Figs 4B and 6B). The successful resection of the ACL was verified in the MRI scans (Fig 8). However, no subchondral edema could be detected and the cartilage layer was thin in the assessed regions. The ACLR medial and lateral condyle showed a MRI-score of 0.75 ± 1.39 and 0.38 ± 0.7 while the ACLR lateral tibia plateau showed a score of 0.25 ± 0.66 and the medial tibia plateau a score of 0. On the sham side, the medial condyle showed a score of 0.13 ± 0.33, whereas the lateral condyle and the medial/lateral tibia plateaus showed a score of 0. No correlation was evident between the histological and MRI scores (r = 0.10021). Also after unblinding the samples, closer examination of the histological findings and MRI scoring showed concurrence in the detected degeneration signs (Figs 3 and 6) in some cases, but in others no concurrence was found (Figs 4 and 5).

**Fig 9:**
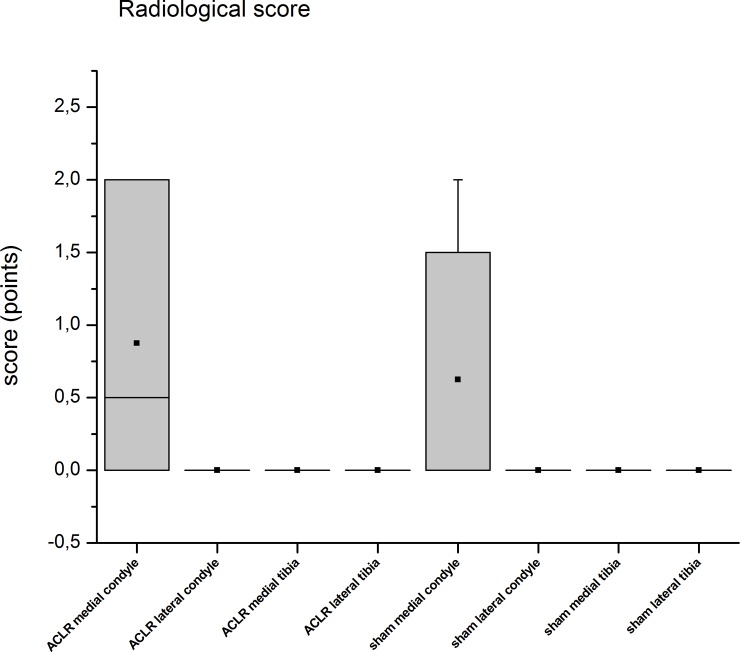
Radiological grading. Radiological grading (from 0 to 4) according to the Kellgren and Lawrence Score [32]. The examined areas of the ACLR and sham sites showed similar findings in terms of degeneration. This also supported our assumption that the sham-operated knee joints may not qualify as a reliable control group. Another aspect of the x-ray analysis was that only the medial condyles of the ACLR and sham sites showed changes of any kind. This would indicate that changes would be initially starting in the medial condyle.

The x-ray assessment revealed signs of degeneration for the medial condyles of either the ACLR or sham knee joints Fig 9. There were no distinct differences between the left and right knee joints. Examination of the narrowing of the joint cavity was not possible, as the legs of the animals had to be extended manually. In some of the ACLR knee joints osteophytes had formed.

**Fig 10:**
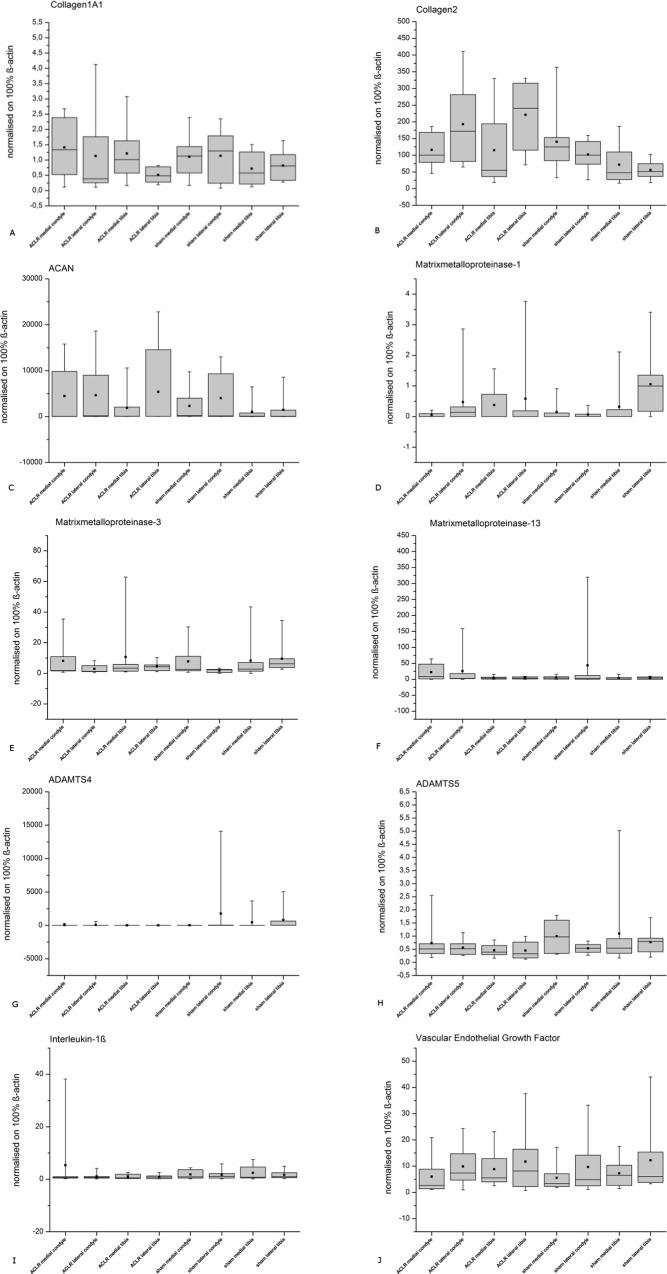
Gene Expression. Normalized (ß-actin) gene expression for the evaluated genes from the examined regions of ACLR and sham knee joints. The results showed no evident differences between the ACLR and the sham operated side for the assessed genes A) *col1A1*, D) *mmp1*, E) *mmp3*, F) *mmp13*, H) *adamts5*, I) *il-1ß* and J) *vegf*. This would concur with the histological findings, where the sham operated group was as degenerated as the ACLR group and therefore not usable as a serious control group. The gene expressions of B) *col2*, C) *acan* and G) *adamts4* at first sight appeared to be differently expressed but as standard deviation was high, significant differences were not found. So no significant differences between the ACLR and sham sites were found in terms of gene expression in the end and therefore, no discrimination between an induced (ACLR) and spontaneous (sham) OA could be made.

No evident changes in the gene expression profiles were obvious for any of the tested genes, neither in the ACLR nor in the sham group. As the results showed small differences in the mean and high standard deviations, no significant differences were assumed at all (Fig 10). No correlation between the histology and gene expression was seen (r-values Table 2).

**Table 2:** Correlation gene expression vs histology. Correlation (r-values) of the gene expression findings with the scored histological findings.

Name	r-value
Collagen 1A1 (*col1A1*)	0,20395
Collagen 2 (*col2*)	0,14545
Aggrecan (*acan*)	-0,19936
Matrix metalloproteinase 1 (*mmp1*)	-0,09115
Matrix metalloproteinase 3 (*mmp3*)	-0,07496
Matrix metalloproteinase 13 (*mmp13*)	-0,06827
ADAM metallopeptidase with thrombospondin type 1 motif, 4 (*adamts4*)	-0,17432
ADAM metallopeptidase with thrombospondin type 1 motif, 5 (*adamts5*)	0,03295
Interleukin 1ß (*il-1ß*)	-0,17442
Vascular endothelial growth factor (*vegf*)	-0,12195

**Fig 11:**
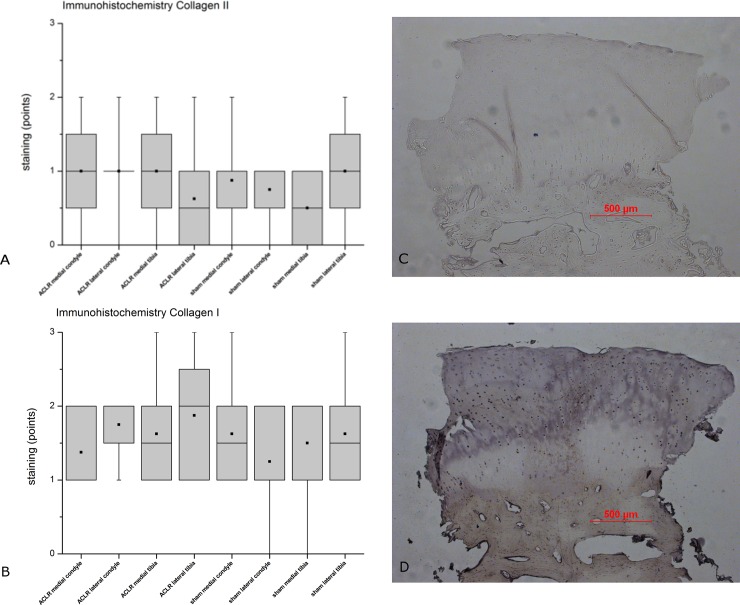
Immunohistochemical staining. Results of the immunohistochemical staining of the examined regions of ACLR and sham operated knee joints with A) collagen II and B) collagen I. The score ranges from 0 (no staining) to 3 points. The results indicated no evident differences between the ACLR side and the sham side for neither Col II (A) nor Col I (B). This was in concurrence with our histological results (Fig 2). Sample of an immunhistological staining of collagen II (C) and collagen I (D) from the moderate degenerated (histological score: 9 points) sham operated side of the lateral tibia plateau of animal #8952 with an intense staining for collagen I (3 points) and a weak staining for collagen II (1 point).

The immunohistochemical analysis of collagens I and II of the samples is shown in Fig 11. Analogous to the histology and gene expression, no differences between the ACLR and sham knee joints for either collagen I or II were found. Neither could a correlation between the histological and immunohistochemical findings be found (r = 0.11607 for collagen I and r = 0.07921 for collagen II).

## Discussion

This study was aiming to induce osteoarthritis (OA) in Göttingen Minipig (GM) through the resection of the anterior cruciate ligament (ACLR) according to Pond and Nuki [11]. The resection of the ACL rather than a transection was chosen to prevent a possible self-healing of the ligament, which was described by Costa-Paz et al. [46]. The findings were then to be verified by histological and MRI scoring as well as an analysis of gene and protein expression. Although the Pond-Nuki model is widely used with dogs [12,47,48], rabbits [1,49], cats [50], lapines [14] or rats [4,51], the model seems to be rarely used with GM, which is a common large animal model in articular cartilage research [5,17,52,53]. The Pond-Nuki- procedure with minipigs was described recently by Wei et al. [6] and the same model was used in Yucatan minipigs [5,54], the latter to study anterior cruciate ligament healing but not degeneration of the articular cartilage. To our knowledge, most animal models for degeneration of the cartilage are models in which a secondary osteoarthritis is induced either by creating an instability in the knee joint by ACLT or ACLT plus menisectomy [1,3,55,56,57], mechanically damaging the cartilage as groove model [3] or damaging the cartilage via chemicals e.g. by monosodiumiodoacetate [58]. Several small animal models for spontaneous OA are known [58,59], a common one is the model with Dunkin Hartley guinea pigs that develop spontaneous OA dependent on age [60]. With large animals, models of spontaneous OA exist as well, e.g. with dogs [61], that highlight the influence of age and breed, and macaques [62]. It was reported that the medial tibial plateau was the most severely affected site [62,63], which was only partially consistent with our findings (Fig 2). Unfortunately, the expression patterns in our study show no indication of the occurrence of spontaneous or induced OA.

The animal models for inducing a secondary osteoarthritis mainly use the histological “Mankin-score” [64] or a modified version [65,66,67,68] to score the degeneration of the cartilage. The original histopathology score was devised by the OARSI histopathology initiative [69] to evaluate OA histopathology. The follow-up initiative (2010) devised species specific consensus scoring systems [35,70,71,72,73,74,75] which are reasonably easy to use and can be readily adopted. There was no specific score available for the GM, thus the OARSI scoring system of Little et al. 2010 [35] for sheep and goats was used for the supposed comparability of the GM including size.

In literature, untreated knees are reported to have mostly a zero or very low mankin-score [55,76,77] but reports of higher scores are also published [78,79]. The results depend also on the area the cartilage was taken from, e.g. the medial or lateral, condyle or the tibia plateau [78,79]. It is reported that treated knee joints often revealed a higher level of degeneration than untreated knee joints [1,4,41,55,66,76,77] but some [78,79] showed similar levels of degeneration.

In the presented study, the analysis of the histopathology showed variations in the level of degeneration in all parts of all knees, as was also shown by Lorenz et al. [12]. The different levels of degeneration in our study were represented by fissures in variable length and quantity, by variable staining, a disparate chondrocyte density, differing cell cloning and tidemark integrity. However, we found no significant differences between the ACLR- and sham-operated joints unlike Frost-Christensen et al. [3]. But Frost-Christensen et al. [3] also resected the medial meniscus and therefore added an additional trauma. The unexpected similarities between ACLR- and sham-operated knee joints may have different explanations. The BW may be a risk factor to develop OA as discussed by [80,81] also for metabolic factors. The GM used in the presented study had a mean BW of 55kg at an age of approx. 24 months. The breeder of the animals (Relliehausen Experimental Farm, Dassel, Germany) reports a BW of 35–40kg for 24 months old GM of as normal [82]. The used animals in the presented study showed a 1.37 fold higher weight than assumed. Thus, the enhanced BW may contribute to the development of the OA in the pigs in both knee joints. Another reason might be the fact that the animals did not move around extensively during their life time in the present study. But as Miyataka et al. [83] showed, even forced running had small effects on cartilage degeneration in mice and Moriyama et al. [84] reported no degenerative changes due to exercise in rats. Cruz et al. [85] showed that a 20min of uninterrupted daily exercise had no effect on the joints of pigs but with additional obstacles it did. Elsaid et al. [86] found that the exercise of joints in rats resulted in an increased cartilage degeneration. Thus it is not clear if exercising may induce or accelerate degenerative changes in articular cartilage. Another problem with exercise in pigs may be that swines are hardly adapted to an exercise protocol or to protect weight-bearing as stated by Chu et al. [87]. Yet another explanation might be that the resection of only the anterior cruciate ligament and the imbalance of kinetics in the knee did not have the expected impact on the induction of osteoarthritis [88] in articular cartilage. However, in our study the sham-operated knee joints also showed signs of degeneration, which is at least in parts contrary to the findings of [89,90,91,92]. But one can assume that the sham site was exposed to higher forces as the contralateral ACLR site was protected. This could have induced degeneration by overstress leading to a secondary degeneration at the sham–operated side. What is more, the sham–operation itself could have induced changes in the joint [92], even though the inner structures were not altered through surgery despite arthrotomy and irrigation [89,90,91]. Thus it seems unlikely, that the sham–operation itself may be the cause of the higher degrees of change in the articular cartilage. Further studies [93,94,95,96,97] were able to show that the cartilage of sham operated animals was not distinguishable from non operated controls in terms of degenerative changes. Neither Calvo et al. [93] nor McDevitt et al. [96] could find significant differences between the non operated controls and the sham operated knees in their animal models with rabbits and dogs respectively. This was also confirmed later for a large animal model with sheep by O’Brien et al. [97]. Therefore the inclusion of a control group of animals without surgical treatment seemed not to be justified when the study protocol was performed also in terms of animal welfare (http://eur-lex.europa.eu, European directives on the protection of animals used for scientific purposes, DIRECTIVE 2010/63/EU, visited July 25th 2016 and [98]).

Yet another risk factor is age. GM reach their skeletal maturity with the closure of the growth plates which takes place between 18 and 22 month of age ((http://minipigs.dk, visited July 25th 2016; cited also by Chu et al. [87]). As growing pigs were shown to possess a higher intrinsic cartilage repair potential [99] they should be skeletally mature. On the other hand they should not be much older to reduce the danger of degenerative changes through to age [100]. These degenerative changes through aging were also addressed in human cartilage [101,102].

A further explanation for the changes in the sham-operated knee joints may be that each operation triggers an inflammatory response of the body which could lead to degeneration of the cartilage [103,104]. In the course of this, proinflammatory cytokines like IL-1ß are known to induce production of MMP´s which in return are degrading components of the articular cartilage [104]. Aigner et al. [18] on the other hand described a down-regulation of the gene expression of many genes in the IL-1 pathway–including IL-1ß—in primary OA cartilage.

We did not find any differences in the gene expression of IL-1ß in the present study between the ACLR and sham-operated group. Thus, we cannot exclude that the sham operation may have induced a process of inflammation leading to the changes.

It is possible that a more or less developed stage of OA was prevalent in the animals when included in the study. Looking at the results of the gene expression of the assessed markers we were not able to detect any differences that could help to differentiate between the outcomes of the sham and the ACLR side.

MRI serves as an important non-invasive tool to detect OA. Especially the early detection of OA has recently been the focus of several publications [105,106,107]. However, MRI morphology often does not correlate with the clinical outcome after cartilage repair surgery. It could also be that specific parameters that correlate best can vary according to the type of procedure performed [108,109].

Histology is the golden standard [106,107,110] for assessing matrix changes associated with the progression of OA. Hence it is important to work on a validation to correlate image findings in MRI with tissue histology, so histology could be an appropriate *ex vivo* reference for comparison with MRI [107].

Surprisingly, a correlation of the histological scoring with the MRI-scoring by use of the modified WORMS-score showed no concurrence. Kreinest et al. [45] report similar findings, as degenerative changes of the menisci were not detected by MRI also in the same animals as we assessed in the present study.

For the detection of OA with MRI, lots of different sequences are available and used [1,111,112,113]. There is general agreement that cartilage damage evaluation with standard MRI examination remains problematic. However, recently cartilage specific MRI sequences were developed with different degrees in specificity and sensitivity [114,115]. Furthermore, there are significant differences in the detection of OA depending on the severity of the disease. In a study by Bachmann et al. [111], 14 different MRI-sequences were utilized to detect cartilage changes in different OA stages and the results were compared with histology and arthroscopy. Here it was obvious that a severe OA could be detected with SE-sequences up to 94% and mild osteoarthritis could be discovered in 10% of the specimen [111]. In several other studies different MRI sequences were used to detect cartilage degeneration [15,116,117]. It was confirmed that even after MRI examinations with FLASH and DESS sequences low correlation in terms of sensitivity and specificity was found for both sequences in normal to mild Mankin grades [112]. Only moderate to severe changes were diagnosed with higher significance and specificity. The possibility of diagnosing early cartilage changes with high accuracy could not be proven by our used sequences. These results are confirmed by Casula et al. [118], reporting that the loss of cartilage and the quality of remaining tissue in the lesion site is not directly associated with each other, because quantitative MRI parameters (especially T1 relaxation time, T2 relaxation time, and delayed gadolinium-enhanced MRI) were not linearly related to arthroscopic grading in OA.

On the other hand, other studies show that quantitative MRI is able to detect early osteoarthritis changes as classified by the OARSI histological system [106]. Streitparth et al. [119] and Tsai et al. [120] reported a correlation between the histological grading and the MRI results. So it is obvious, that there is a variety of studies which work with different parameters, so that the results cannot be compared. Another aspect is the different intra-articular anatomical geometry between the porcine and the human knee and the difference in size. So there may not be comparability between human and porcine studies. Furthermore, clinical MRI diffusion protocols are seriously affected by various artefacts among them geometrical distortion and susceptibility effects [105]. In our case, it is possible that the histological and corresponding MRI regions may have been marginally different. This is related to a potential mismatch in plane orientation and disproportion in image resolution. In the present study MRI slice thickness of 3 mm has to match a histological osteochondral plug of 4 mm in diameter. However, after unblinding the samples, we compared and tried to allocate the areas of histological changes to the MRI scans and vice versa but did not always find a match in the MRI. Saadat et al. [16] showed that cartilage thickness and surface lesions could be assessed in human cartilage by the MRI sequences but that signal changes of the cartilage were not suited to indicate the amount of cartilage degeneration. Fischbach et al. [121] and Wachsmuth et al. [122] however reported difficulties in detecting cartilage lesions with MRI. This may explain the lack of a match between our histological and MRI findings, because the structure of the cartilage, especially lesions and fissures, is a main “point contributor” to the histological Little score [35]. On the other side, fissures could not sufficiently be detected by the MRI in our study, so its contribution to the MRI score was insignificant. The fact that the articular cartilage of the Minipig knee joints used in our study is thinner [87,123] than the cartilage of human knee joints [123,124] may be another explanation for the difficulties in finding the discovered histological degeneration in the MRI images. Wei et al. [6] showed differences between the control group and the treated group in quantitative MRI analysis by delayed gadolinium enhanced MRI of cartilage with the dGEMRIC technique over a time period of 2 to 6 weeks with a positive correlation of the GAG content to T1, Gd values and negative correlation to T2 values [6]. The blinding of the MRI scans may not avoid bias of the evaluators as the ACL was visibly absent after resection in the scans (Fig 8). Though this was a good validation of a successful surgery, an unbiased assessment of the MRI scans was hardly possible. This may be reflected by the higher scoring of the left condyles, both medially and laterally (Fig 7). However, this problem can hardly be avoided as the ACL as an area of interest is an inner anatomical structure of the stifle joint close to the articular cartilage. The radiological assessment of the knee joints showed no severe signs of degeneration. This may be due to the fact that the x-ray examination was done by manually pulling the limb to get a good view of the joint space. Therefore the examination of the narrowing of the joint cavity as described by [32] was not possible, which is an important factor in the Kellgren and Lawrence score [32].

A literature search (n = 81) for gene expression profiles of osteoarthritic and normal cartilage was performed for the genes which were to be assessed in this study. The identified genes were assessed in terms of their expression level. As the descriptive statistic shows, there are no evident changes in expression patterns between the two groups (Fig 10).

The results of the gene expression profiles in our study differ from the findings of other authors who for example showed an enhanced expression of *col2* [18,21,125] or of *col1a1* [12,18,21,22,66,125,126,127] in degenerated cartilage. However, *col1a1* was upregulated in the menisci of the animals used in the presented study which was identified as degeneration of the meniscal tissue [45]; Thus a change in the assessed articular cartilage should have been detected. For other genes, for example *acan*, our findings were shared with some authors but differed from the findings of others [12,18,21,41,125,127,128,129]. The lack of difference in the gene expression results between the ACLR- and sham-operated groups in our study could be expected as it is in accordance with our histological findings. Due to that, we cannot discriminate between *verum* and control group in terms of gene expression as the control group (sham operated) showed similar degenerative changes of articular cartilage to the ACLR-group.

As in our study the sham-operated site also developed OA, we looked at the expression patterns of genes thought to be involved in the OA process that caused the differences between the sham and ACLR joints. Different levels of gene expression could help to identify genes relevant for the development of spontaneous (sham-site) or induced (ACLR-site) OA. But as there were no differences, we could not distinguish between a spontaneous or induced OA through the gene expression profiles.

An obstacle in the present study was the small amount of obtained cartilage tissue for gene expression profiling, which was done in duplicates only; however, several studies used a similar approach [130,131,132].

In our study, distribution of collagen I and II in ACLR and sham knee joints was variable for all included animals and did not correlate in any way with the histological findings. A variability of collagen II distribution in animals was also seen by Bernstein et al. [23]. Another publication by Barley et al. [133] reported an increased staining of collagen II and a decreased staining of collagen I in normal cartilage. This may be due to the fact that in our study the standard deviation was high and the sample number was low and therefore further studies need to be performed. The fact that the results showed high standard deviations may indicate a high individual variability of the animals in terms of degeneration grade. Therefore an increase in the number of animals may provide more valid results. On the other hand the *verum* (ACLR) and the control (sham) knee joint originate from the identical individual, so that the inter-individual variability of the animals is lessened. Our study design requested a life time of the GM for 26 weeks after the ACLR operation to maximize possible degenerative effects. Other authors used shorter time spans of up to 14 days [5] or up to 6 weeks [6] but with other species, longer life time [11] were used. A longer life span with an unbalanced knee joint is expected to raise the risk of developing OA or worsen an already existing condition in terms of the histological/radiological findings. However, the time of observation over 26 weeks revealed OA on both the ACLR and the sham side.

An increased expression in the RNA analysis is not necessarily associated with an increased protein level [134,135,136]. So the absence of protein detection of most of the examined genes may be a limitation of the presented study. However, most relevant proteins Col II and Col I were examined by IH (Fig 11C and 11D) showing no significant differences between the sham and *verum* group (Fig 11A and 11B) as was also seen in the gene expression profile (Fig 10B and 10A). One has to consider that further investigation of the proteins (e.g. Western Blotting) is material consuming. The amount of material of articular cartilage in the presented study was limited. According to the protocol (see above) cartilage tissue was available originating from an area with 10 mm in diameter minus the middle region of 4 mm in diameter serving for the histological examination. The aim of this procedure was to gain samples for the histological and the gene expression in immediate vicinity of each other. Therefore it was not possible to extend the range of the harvesting area.

## Conclusion

OA was detected by histological findings in both knee joints of Göttingen Minipigs after 26 weeks regardless of the intervention. MRI results did not match the results of scoring the degenerative histological changes. Furthermore, the gene expression analyses of 10 promising genes did not help to answer the question, whether the degenerative changes may be induced or idiopathic. Further studies analyzing the articular cartilage may reveal differences between sham operated knee joints and after ACLR at the protein level. Regarding the findings in the presented study the resection of the ACL does not seem to be an effective method to induce isolated OA in adult minipigs.

## Supporting Information

S1 Table Raw data. (XLSX)
